# Mesenchymal stem cell therapy: A review of clinical trials for multiple sclerosis

**DOI:** 10.1016/j.reth.2022.07.003

**Published:** 2022-08-23

**Authors:** Asma Alanazi, Mohammad Alassiri, Dunia Jawdat, Yaser Almalik

**Affiliations:** a College of Medicine, King Saud Bin Abdulaziz University for Health Sciences, Riyadh, Saudi Arabia; b College of Science and Health Professions, Riyadh, Saudi Arabia; c King Abdullah International Medical Research Center, Riyadh, Saudi Arabia; d Division of Neurology, King Abdulaziz Medical City, National Guard Health Affairs, Riyadh, Saudi Arabia

**Keywords:** Multiple sclerosis, Mesenchymal stem cells, Hematopoietic stem cells, Stem cell therapy, Regenerative medicine

## Abstract

Multiple sclerosis (MS) is a disease of the central nervous system (CNS) that is the result of the body's own immune cells being auto-reactive to the myelin regions of the body as if these regions were foreign antigens. This demyelination process is damaging to the electrical conductivity of neurons. The current medicines are only capable of fighting off the symptoms of the disease, but not the disease itself. Specialized stem cells, known as mesenchymal stem cells (MSCs), seem to be the candidate therapy to get rid of MS. MSCs can be isolated from multiple sources of the person's body, and even from the umbilical cord (UC) and placenta of a donor. These cells have anti-inflammatory effects so they can target the overactivity and self-antigen attacks by T cells and macrophages; this immune system overactivity is characteristic of MS. MSCs show the ability to locate into brain lesions when injected and thus can compensate for the loss of the brain function by differentiating into neuronal precursor cells and glial cells. The author has listed tables of clinical trials that have utilized MSCs from different sources, along with the years and the phase of study completed for each trial. The consensus is that these cells work on inhibiting CD4^+^ and CD8^+^ T cell activation, T regulatory cells (Tregs), and macrophage switch into the auto-immune phenotype.

The best source of MSCs seems to be the UC due to the easiness of extraction, the noninvasive method of collection, their higher expansion ability and more powerful immune-modulating properties compared to other locations in the body. Studies showed there was a significant decline of mRNA expression of several cytokines after the administration of MSCs derived from the UC (UCMSCs). Other researchers were able to repair the defects of Tregs in MS patients by co-culturing Tregs from these patients with UCMSCs, which decreased the production of the pro-inflammatory cytokine IFN γ, and also suggested a strong link between Tregs lack of functionality in MS patients with the pathogenesis of the disease.

## Abbreviations

AD-MSCs: Human adipose-derived mesenchymal stem cells
AICD: Activation-induced cell death
ALS: Amyotrophic lateral sclerosis
APCs: Antigen-presenting cells
ASCs: Adipose-derived stem cells
BAEP: Brainstem auditory evoked potential
BH: Black hole
BBB: Blood–brain barrier
BM-MSCs: Bone marrow-derived mesenchymal stem cells
CNS: Central nervous system
CSF: Cerebrospinal fluid
CTL: Cytotoxic T cells
DCs: Dendritic cells
EAE: Experimental autoimmune encephalomyelitis
EDSS: Expanded disability status scale
Gd: Gadolinium
GEL: Gadolinium-enhancing lesions
IFN γ: Interferon gamma
IL: Interleukin
MHC: Major histocompatibility complex
MRI: Magnetic resonance imaging
MS: Multiple sclerosis
MSCIMS: Mesenchymal stem cells in multiple sclerosis
MSCs: Mesenchymal stem cells
ODCs: Oligodendrocytes
PBMCs: Peripheral blood mononuclear cells
PPMS: Primary progressive multiple sclerosis
RRMS: Relapsing-remitting multiple sclerosis
SEP: Somatosensory evoked potential
SPMS: Secondary progressive multiple sclerosis
SSS: Sexual satisfaction scale
TCR: T-cell receptors
Teffs: T effector cells
TGF: Transforming growth factor
Th: T helper
TNFα: Tumor necrosis factor
Tregs: T regulatory cells
UC: Umbilical cord
UCMSCs: Umbilical cord mesenchymal stem cells
VEP: Visual evoked potential

## 1 Background on multiple sclerosis

Multiple sclerosis (MS) is a chronic, demyelinating, autoimmune-mediated neuroinflammatory disease of the central nervous system (CNS). MS immunopathogenesis involves CNS inflammation, blood–brain-barrier (BBB) disruption, and attacks of neurologic symptoms that often lead to limb paralysis, serious problems in sensation, and partial or complete loss of central vision, fatigue, dizziness, lack of sleep, and depression [[13], [20]]. MS is categorized into several phenotypes: primary progressive MS (PPMS), relapsing-remitting MS (RRMS), and secondary progressive MS (SPMS) [64]. The progressive phase is characterized by an irreversible neurodegeneration and axons damage, but the incidence of RRMS is higher than the other types and is associated with worsening attacks of neurological function due to flare-ups of neurological disabilities from time to time [4].

A key pathological feature of MS is an autoimmune mechanism in which auto-reactive myelin-specific CD4^+^ T cells – T helper (Th) cells – target the self-antigens of the myelin in the CNS after penetrating the BBB, in an uncontrolled response of the immune system. Neurons transmit information by electrical signaling and to do this efficiently, their axons should be insulated with myelin. Oligodendrocytes (ODCs) are cells that provide support and insulation for axons by forming the myelin sheath. The auto-reactive T cells initiate and propagate an autoimmune response against the CNS, and in the process attack myelin, ODCs, and neurons. Death of ODCs is the primary causes of axonal loss, demyelination, and damaging of CNS by the formation of CNS plaques [15]. CNS plaques are composed of inflammatory cells and their products, demyelinated and transected axons, and astrogliosis in both white and gray matter [37].

It is known that some major histocompatibility complex (MHC) haplotypes, as well as some alleles of cytokines and their receptors, increase the risk for MS (Gourraud et al 2012). An interaction between environmental and genetic factors in the susceptibility to MS has been suggested by recent findings, offering possible scenarios for collaboration between specific factors in MS initiation. For instance, MS risk modulators, including genetic variants in interleukin-7 receptor-α (IL7RA∗C), IL-2 receptor-α (IL2RA∗T), MGAT1, and CTLA-4, and environmental factors affecting vitamin D3 levels, converge in order to alter branching of Asn (N)-linked glycans [44], Gourraud et al 2012. More importantly, branching reduction results in T-cell hyperactivity and promotes spontaneous inflammatory demyelination and neurodegeneration in the MS animal model. N-glycan branching is positively regulated by Mgat1 and Mgat5, both of which are Golgi body enzymes. Mgat1 is down-regulated by IL7RA∗C; IL2RA∗T is opposed by Mgat1 and vitamin D3, but IL2RA∗T optimizes branching and mitigates the risk of MS when combined with enhanced N-glycosylation of CTLA-4. Therefore, various genetic and environmental factors regulate a final common pathway: N-glycosylation. This pathway is highly relevant for MS pathogenesis [17].

[17] and [50] have shown that vitamin D directly stimulates the expression of HLA-DRB∗15, MHC class II allele, which is a major MS genetic risk factor [18]. suggested that a low vitamin D level in early childhood leads to a decrease in the expression of HLA-DRB∗15 in the thymus, which results in an inadequate presentation of self-antigens. Consequently, more auto-reactive T cells could emerge in the immune system of people who had low vitamin D in their early childhood. Below is a sketch that represents the major cell types of the CNS and the immune system in the pathogenesis of MS disease [38].

T-cell activation, which can result in the attack on self-myelin, comes from the recognition of antigens presented by MHC molecules on the surface of antigen-presenting cells (APCs). Dendritic cells (DCs) are APCs that are capable of activating naïve T cells, which makes DCs central in the initiation of the adaptive immune response [9]. T cells have T-cell receptors (TCR) and co-receptor molecules (CD4 or CD8) that help them bind with MHC complexes on the molecules of APCs. If a T cell expresses CD4, it recognizes MHC class II molecules, and if it expresses CD8, it recognizes MHC class I molecules. T cells that express CD4 are designated as CD4^+^ T cells, and those that express CD8 are designated as CD8^+^ T cells, and are known as cytotoxic T cells (CTL). Th cells produce mediators that perform effector and regulatory functions in immunity, while CTL cells kill infected, or damaged, cells. Th cells are integral to the pathogenesis of MS as they play a critical role in the maturation and complete activation of other immune cells. Th cells can be differentiated into Th1, Th2, or Th17 phenotypes when Il-2, IL-23, or IL-4 bind to them. Once differentiated, the Th1 phenotype releases interferon gamma (IFN γ) and tumor necrosis factor (TNF-α), both of which are proinflammatory cytokines that can promote inflammation by suppressing Th2 differentiation, and thus canceling the Th2 release of IL-4 and IL-13, both of which are anti-inflammatory cytokines [79]. Th17 is another type of CD4^+^ T cells that induces a large number of cytokines, including IL-17, IL-21, IL-22, and IL-26, all of which are capable of promoting inflammation (Ouyang et al 2008).

Without the support of other Th cells, B cells do not mature into long-lasting memory cells or plasma cells. CTL are also not fully activated without Th cell involvement. CTL can also be found in MS lesions, according to many studies, as outlined by [31]. CTL can trigger ODCs death and thus play an important role in MS pathogenesis. Once the ODCs are impaired, the myelin repair process is also impaired [27]. There are several types of effector Th cells, which include Th1 and Th17, that appear to be major pathogenic populations in MS [39]. Once T cells enter the CNS, they will be nonfunctional unless reactivated upon their entry. While there are no DCs in a healthy CNS [51], other APCs are capable of reactivating T cells in the CNS. Besides macrophages, CNS contains other semi-professional APCs, which include microglia, B cells, and endothelial cells, as well as nonprofessional APCs (astrocytes), which can reactivate T cells [1].

In the process of T cell maturation, T cells that recognize self-antigens are eliminated or inactivated by mechanisms of central and peripheral tolerance. Central tolerance is based on the interaction between TCR and MHC, which takes place in the thymus. A naïve T cell can be activated only if it receives simulation simultaneously through TCR, coreceptors, and cytokines. If the recognition lasts too long, or is too intense, a T cell will be eliminated in the process of activation-induced cell death (AICD) [16].

In a healthy individual who does not suffer from MS, Tregs play a crucial role in self-tolerance control and peripheral tolerance, by dynamically suppressing the activation and expansion of auto-reactive T cells and other immune cells in peripheral and CNS systems. Tregs do this by secreting immunosuppressive mediators through cell-to-cell contact, and also via the inhibition of the stimulatory capacity of APCs [29,55]. It is hypothesized that the reduced number of Tregs in MS patients is responsible for the activation of auto-effective T cells in the peripheral and CNS These auto-effective T cells initiate and propagate an immune system attack which damages the CNS via demyelination and killing of ODCs [32,23].

Many studies report that the adoptive transfer of autologous or allogenic Tregs reversed, and even inhibited, autoimmune disease development; their transfer was not accompanied by any immune systems as these cells controlled the allo-immune response to organ and cell transplantation by cell-to-cell contact, secretion of anti-inflammatory cytokines, and modulation of APCs [34,78]. Pre-clinical and clinical studies have shown that autologous Tregs may have potential as a novel therapy in the treatment of autoimmune diseases. However, other studies have confirmed that Tregs that are in the patients who have autoimmune diseases like MS, cannot efficiently inhibit the proliferation of auto-effective T cells as Tregs in these patients have become impaired in their suppressive function [67,68]. Therefore, developing clinically applicable protocols that reverse or repair the impaired suppressive function of these Tregs would be necessary before autologous Tregs are to be used as real therapeutic cells.

Currently, there isn't an effective therapeutic model for MS disease. Current medications are costly and are focused on lessening the symptoms and chronic inflammation, but not curing the disease or repairing the damaged myelin. Furthermore, the symptoms were recurrent or became aggravated after drug withdrawal. Adding to this is the unfortunate reality that medicines with immunomodulatory and immunosuppressant properties provide partial efficacy to ameliorate autoimmune reactions. This is the reason why disease progression can lead to approximately 50% of affected patients to develop chronic progressive disease with a poor prognosis (Lei et al 2014). Recent evidence has suggested that an appropriate treatment should be centered on the modulation or suppression of aggressive immune response, protection of neurons and axons against degenerative process, as well as improvement of repair or remyelination [14].

## 2 Mesenchymal stem cells

MSCs are multipotent adult stem cells that are highly proliferative, are capable of self-renewal, and have immunomodulatory and neurodegenerative effects [61]. They can also attenuate overactive immune and hyperinflammatory processes as they are capable of inducing Foxp3+ expression in CD4 T cells *in vitro* [10]. In addition, MSCs display multiple regulatory roles in the immune system by inducing the generation of Tregs via cell–cell contact between MSCs and T cells, and also by secreting anti-inflammatory factors *in vitro*, which would allow them to control the progress of autoimmune diseases, including MS [62,60,72]. Their immunomodulatory actions may be exerted by a direct contact with immune cells or by paracrine activity. It is reported that MSCs can inhibit Th17 and Th1 cell differentiation [58,24]. They can also promote repair and regeneration of tissue by having the ability to differentiate into multiple cell types. Moreover, they can also antimicrobial molecules and they do so with low immunogenicity (low levels of class I and class II human leukocyte antigen). Previous studies have suggested that MSCs amplified *in vitro* can suppress the proliferation of T lymphocytes, B lymphocytes, and natural killer cells, and can also inhibit maturation and differentiation of dendritic cells [28,60].

MSCs can be easily isolated from peripheral blood, bone marrow, adipose tissue, umbilical cord (UC), and placenta [5,33]. Later, they can be expanded in culture media to create a large population of cells for cell-based therapy [49]. Adding to the easiness of their extraction, a patient can also be served as a donor for himself without the risk of rejection, and it has been found that autologous MSCs carry a safer pattern without the risk of malignant transformation [[53], [71]].

Stem cell-based therapy has recently provided a hope for treating MS patients, and is now considered the most preferred and noninvasive method for treatment of different diseases [71]. Experimental studies showed that intravenous administration of MSCs has immune suppressive effects and ameliorates autoimmune diseases [25]. It has also been shown that MSC transplantation significantly improves the clinical outcomes of MS in experimental autoimmune (EAE) models [3]. Up-going and completed clinical studies that investigated the effectiveness and safety of MSCs in MS treatment revealed that upon intravenous injection into the cerebrospinal fluid (CSF) of the spinal cord, MSCs are able to locate into brain lesions. This should improve the survival rate of brain cells via the cells’ differentiation into neuronal precursor cells and glial cells, thereby compensating for the lost brain function. This could decrease disease severity and improve the quality of life in patients with MS [69,46]. Table 1 discusses the years, countries, and phases of study for the application of autologous MSCs in cell therapy, which are derived from oneself so would not present the risk of rejection and cannot possibly become malignant (see Table 1).

**Table 1:** Mesenchymal stem cells therapy and clinical trials for MS disease.

Cell Type	Years	Country	Phase	Evaluation after Cell Therapy
Autologous MSCs	2013	Italy	1 & 2	MSC therapy without side effect infusion (NCT01854957)
Autologous MSCs	2011–2018	Iran	1 & 2	Evaluate the effect of MSC transplantation on number of Gd (gadolinium)-positive lesions (NCT01377870)
Autologous MSCs	2013–2016	UK	1 & 2	MSC therapy without side effect after infusion, quantified by the reduction in the number of new contrast-enhancing lesions on MRI scans (NCT01606215)
Autologous MSCs	2010–2014	Spain	2	No identification of any serious adverse events, at 6 months, patients treated with MSCs had a trend to lower mean cumulative number of GEL (gadolinium-enhancing lesions), and at the end of the study to reduced mean GEL, non-significant decrease of the frequency of Th1 (CD4^+^, IFN $\gamma^{+}$) cells in blood of MSCs treated patients (NCT10228266)
Autologous MSCs	2014–2018	Canada	2	Efficacy: total number of GEL on MRI scan (NCT02239393)
Autologous MSCs	2011–2016	USA	1	Evaluated the feasibility of culturing MSCs, and infusion-related safety and tolerability of autologous MSC transplantation over one month in patients with relapsing forms of MS. Stem cell therapy was without side effects (NCCT00813969)
Autologous MSCs	2010–2015	Spain	1 & 2	Evaluated safety and tolerability related to the intravenous infusion of autologous mesenchymal stem cells. Evaluated effects on MS disease activity as measured by: clinical variables, immunological and neurophysiologic analysis, neurophysiological and quality of life scales (NCT01056471)
Autologous MSCs	2001–2005	USA	1	MSC therapy is safe without side effects after injection (NCT00017628)

## 3 Bone marrow-derived MSCs (BM-MSCs)

Bone marrow tissue is an important source of MSCs and these cells have the ability to differentiate into several cell types due to their multi-potential properties BM-MSCs were the first cells to be discovered, which is why a lot of studies used bone marrow as a source of MSCs. They can be administered either through the intraperitoneal route or intravenously. Administration through the intraperitoneal route improved experimental autoimmune encephalomyelitis (EAE), as demonstrated by the amelioration of clinical score and the reduced inflammatory infiltration and demyelination of the spinal cord. These cells inhibited CD4^+^ and CD8^+^ T cell activation, induced Tregs, and modulated macrophage polarization from M1 to M2 phenotype which led to a reduced production of pro-inflammatory cytokines [73]. Table 2 lists several clinical trials that evaluated the effectiveness of BM-MSCs in MS disease treatment; the cells were administered intravenously, a method that has proved to be safe. BM-MSCs that were administered intravenously improved neurobehavior outcomes and reduced inflammatory infiltration as well as demyelination in the spinal cord. The results were centered on the improvement in disease severity, cognitive function, and quality of life of MS patients, thanks to the cells’ neuroprotective and anti-inflammatory properties [7].

**Table 2:** BM-MSCs clinical trials for MS disease.

Cell Type	Years	Country	Phase	Evaluation after Cell Therapy
Autologous BM-MSCs	2014–2016	Israel	2	Changes in immunological response at 12 months following treatment. Neurological function test and Expanded Disability Status Scale (EDSS) improvement (NCT02166021)
Autologous BM-MSCs	2015–2018	France	1 & 2	Primary outcome is safety cell therapy without side effects. Efficacy assessed by combined unique magnetic resonance imaging (MRI) activity, volume of GEL, and volume of BH (black holes) (NCT02403947)
Autologous BM-MSCs	2006–2011	UK [8,7]	1 & 2	Safety and feasibility of the intervention and informing design of future studies to address efficacy, mesenchymal stem cells in multiple sclerosis (MSCIMS) adopts a novel strategy for testing neuroprotective agents in MS – the sentinel lesion approach serving as proof of principle for its future wider applicability (NCT00395200)
Autologous BM-MSCs	2013–2017	Jordan	1 & 2	Patient with any relevant side effects observed, assessing the safety of autologous MSCs injection (NCT00781872)
Autologous BM-MSCs	2006–2009	Israel [26]	1 & 2	Safety and migration ability of the injected cells, clinical efficacy. N side effects. Transplantation of MSCs in patients with MS and ALS is a clinically feasible and relatively safe procedure and induces immediate immunomodulatory effects (NCT00781872)
Autologous BM-MSCs	2013–2016	Spain	1 & 2	Safety and efficacy after cell therapy, subsequent flow cytometry: IL-2, 4, 6, IFN-γ, IL-10, TNF-α, or by ELISA: TGF-β and IL-17 (NCT02035514)
Autologous BM-MSCs	2017	Jordan	1	Effectiveness assessment by MRI, safety assessment by physical examination, vital signs, analytical results, electrocardiograph monitoring, and EDSS (NCT03069170)
Autologous BM-MSCs	2001–2005	USA [2]	1	Four new or enhancing lesions were seen on MRI, all within 13 months of treatment. In this population with high baseline EDSS, a significant proportion of patients with advanced MS remained stable for as long as 7 years after transplant (NCT00014755)
Autologous BM-MSCs	2015–2018	Spain	1 & 2	MS therapy is safe without side effects after cell injection. Evaluated EDSS score (NCT02495766)
Autologous BM-MSCs	2013–2018	UK [52]	1 & 2	MS therapy is safe without side effects after cell injection. Evaluated EDSS score (NCT02495766)
Autologous BM-MSCs	2014–2018	UK [52,52]	1 & 2	MS therapy is safe without side effects after cell injection. Evaluated EDSS score (NCT02495766)

## 4 Human adipose-derived MSCs

Human adipose-derived MSCs (AD-MSCs) were able to reduce disease severity when administered both at the onset or during the acute phase of the disease in EAE mice. These cells can be easily isolated by liposuction method from adipose tissue, which is abundant in abdominal tissue and hip area. In addition, they are clinically used in tissue engineering and reconstruction, as well as in cell-based therapies. Experimental studies revealed AD-MSCs can differentiate into myelin-producing cells and compensate myelin loss in MS disease models [30,59]. Both the autologous and allogenic models of ASCs have been frequently used in clinical studies, with several of the studies reporting that the injection of these cells is safe without adverse effects [47]. Table 3 lists the results of several clinical studies that considered the effect of AD-MSCs in MS treatment, with the consensus of it being a safe method that improves MS disabilities such as sexual problems and social activities (see Table 4).

**Table 3:** AD-MSCs therapy clinical trials for MS disease.

Cell Type	Years	Country	Phase	Evaluation after Cell Therapy
Autologous ADMSCs	2018	Spain [11]	1 & 2	Infusion of autologous AD-MSCs is safe and feasible in patients with SPMS (NCT01056471)
Autologous ADMSCs	2012–2015	Sweden [6]	1 & 2	Safety of intravenous (IV) therapy with autologous MSCs in MS patients (NCT01730547)
Autologous AMDSCs	2014–2018	USA	2	Change from baseline in sexual satisfaction at month 12 as measured by participants using the SSS (Sexual Satisfaction Scale) (NCT02157064)

**Table 4:** UCMSCs therapy for MS disease.

Cell Type	Years	Country	Phase	Evaluation after Cell Therapy
Allogenic UCMSCs	2014–2017	China	1 & 2	No clinical attacks occurred during transplantation. MRI revealed a reduced number of foci and EDSS scores were decreased
Allogenic UCMSCs	2017	Jordan	1 & 2	Intensity and volume of CNS lesions assessed to investigate the therapeutic benefits of the injected allogenic MSCs and physical therapy by MRI (NCT03326505)
Allogenic UCMSCs	2014–2017	Panama	1 & 2	Change in disability as measured by EDSS, qualify of life as measured by the SF-36 quality of life questionnaire (NCT02034188)
Allogenic UCMSCs	2010–2014	China [42]	1 & 2	Evaluated core of EDSS, VEP (visual evoked potential), MRI, SEP (somatosensory evoked potential) and BAEP (brainstem auditory evoked potential). No side effects were apparent after cell injection (NCT01364246)
Allogenic UCMSCs	2018	Panama	Not specified	Gadolinium-enhanced MRI scans of the brain and cervical spinal cord were taken at baseline and also 1 year post-treatment. Treatment with UCMSCs intravenous infusions for subjects with MS is safe (NCT0234188) [54]

## 5 Umbilical cord MSCs (UCMSCs)

MSCs extracted from the umbilical cord are termed UCMSCs. They can be easily derived from umbilical cords discarded after delivery, so they do not cause any ethical controversies and their collection method is noninvasive [48]. UCMSCs are bioequivalent to MSCs from the bone marrow. Moreover, UCMCSs are genome-stable, have lower immunogenicity, have higher expansion ability compared to those from the bone marrow and other adult tissues, and have more powerful immune-modulating properties [77,65]. For example, UCMSCs suppressed mitogen-induced lympho-proliferation to a greater extent than bone marrow-derived MSCs. UCMSCs are also capable of promoting the production of Tregs *in vitro* [62] and increasing peripheral Treg *in vivo* [60]. The administration of these cells in cynomolgus monkeys with EAE improved MS clinical symptoms, reduced demyelination and inflammation [35].

UCMSCs can be isolated from different parts of the umbilical cord, such as Wharton's jelly. MSCs derived from this jelly region have high proliferative and therapeutic ability, their administration is of little invasiveness, and lack significant immunogenicity (low levels of class I and class II human leukocyte antigen), which permits allogenic transplantation without immunosuppressive drugs [56,63]. In fact, Mikaeli [43] reported that human Wharton's jelly stem cell-derived oligodendrocyte progenitor cells transplanted into the brain ventricles of an MS mouse significantly decreased the clinical signs of MS and induced functional improvements. Furthermore, histological examinations demonstrated that transplantation of these cells promoted the regeneration of myelin sheaths in the brain lesions.

[36,74] reported that the immunosuppressive function of CD4^+^CD25^+^ Tregs or CD8^+^CD28^—^ Tregs from PBMCs of healthy donors are enhanced *in vitro* by co-culture with allogenic MSCs from bone marrow [54]. have reported on the safety and effectiveness of allogenic UCMSCs injected to MS patients. They found that UCMSCs improved neurological parameters such as the Scripps neurological rating scale, EDSS, the nine-hole peg test, the expanded EDSS rating neurologic impairment, and 25-foot walking time in these patients. Below are several clinical trials on the effectiveness of intravenous injection of UCMSCs in expanded EDSS scores improvement.

[76] carried out a study in which they demonstrated that UCMSCs significantly increased the frequency of CD4^+^CD25^high^CD45RA ^+^ Tregs and the production of anti-inflammatory cytokines in co-cultures with PBMCs from healthy subjects. In another study by [75]; the researchers further investigated the immunomodulatory effects of UCMSCs on the frequency and immunosuppressive function of Tregs from the peripheral blood of MS patients by co-cultures of UCMSCs and peripheral blood mononuclear cells (PBMCs) of MS patients, for 3 days. To repair the defects of Tregs from MS patients, the researchers co-cultured Tregs from MS patients with UCMSCs. It was found that the functional defects of Tregs in MS can be repaired *in vitro* using a simple UCMSCs priming approach as UCMSCs significantly increased the frequency of CD4^+^CD25^+^CD127^low/-^ Tregs in resting CD4^+^ T cells (*P* < 0.01) from MS. Additionally, UCMSCs-primed Tregs of MS patients significantly inhibited the proliferation of PHA-stimulated autologous and allogenic CD4^+^CD25^—^ T effector cells (Teffs) from MS patients and healthy individuals, compared to non-UCMSCs-primed Tregs from the same MS patients (*P* < 0.01). [_Schneider_et_al_2013] suggest that Teffs resistance to the regulation of Tregs may also contribute to the pathogenesis of MS. While [75] did not examine whether there is resistance of Teffs to Tregs, but the data showed that UCMSCs-primed Tregs from MS patients efficiently suppressed the proliferation of autologous Teffs stimulated by PHA, a non-specific stimulant of normal lymphocytes, compared to non-UCMSCs-primed Tregs from the same MS patients. One more significant result to report here is that UCMSCs co-cultures decreased the production of pro-inflammatory cytokine IFN γ.

The study before the last in the table is by [42] who wanted to investigate the clinical efficacy and safety of UCMSCs transplantation for treating two MS patients, for a total of seven times of treatments. The researchers collected UC from healthy pregnant women with no history of infectious, familial, or hereditary diseases. This was done in Yan'an Affiliated Hospital of Kunming Medical University. UCMSCs were isolated and cultured from these cords. The number of cells in each intravenous infusion was 1–2 × 10^6^ cells/kg at 3-month intervals for 7 times. After treatment, clinical effects including symptoms, vital signs, clinical attacks, MRI, neurological function scores, and adverse reactions like fever, dizziness, and vascular irritation were monitored and evaluated.

The research results of [42] did not present obvious adverse reactions or residual pathological syndromes appeared during transplantation. Additionally, the regulatory immunomodulatory effects of UCMSCs on immune system of MS patients were also assessed. This was done by collecting peripheral blood before and after cell transplantation. The results indicated that patients’ symptoms improved after UCMSCs transplantation. No clinical attacks happened during transplantation. MRI revealed a reduced number of foci in both patients, suggesting that UCMSCs transplantation promoted remyelination [22]. Furthermore, the EDSS scores had decreased in both patients, indicating that clinical symptoms were mitigated. Real-time PCR was used to determine the mRNA expression of CD86, interleukin (IL)-2, IL-17c, Foxp3, CTLA-4, and HLA-DRB1, transforming growth factor (TGF)-β 1, and TGF-β 2 in peripheral blood. All but Foxp3, TFG- β 1, and TGF-β 2 were significantly reduced after UCMSC transplantation (*P* < 0.05). The two versions of TGF are responsible for cell proliferation, differentiation, apoptosis, and immunoregulation, while Foxp3 plays an important role in immune protection [21]. The reason for why three immune factors did not decrease after transplantation should be investigated in future studies.

Given that the mRNA expression of each cytokine decreased, this demonstrates the immunomodulatory properties of UCMSCs. CD86 and CTLA-4 are important co-stimulatory molecules with a negative regulating function. When bound together, they produce inhibitory signals. During disease progression, the body's pathological immune response induces high expression of the inhibitory co-stimulatory signals. MSCs correct this pathological immune response *in vivo*, which decreases the expression of CD86 and CTLA-4 [40]. IL-2 is the core of the immune regulatory network [66], whose mRNA expression was reduced after UCMSC transplantation. T-helper 17 (Th17) cells are a subset of CD4-T helper cells, which are characterized by their production of IL-7. IL-7 is a pro-inflammatory cytokine, which is highly expressed in the serum and tissue of patients with rheumatoid arthritis, asthma, systemic lupus erythematosus, and MS [70,12]. In this study, IL-7 expression decreased after transplantation of UCMSC, meaning that UCMSCs inhibited Th17 cell differentiation.

Another detailed study in the table was by that by [54] which included twenty subjects; mean age of enrollees was 41.15 (SD = 9.25) years and 12 of them were females. Fifteen subjects had a diagnosis of RRMS, four had PPMS, and one had SPMS. Enrolled subjects received 140 × 10^4^ UCMSCs intravenously over seven visits (20 × 10^6^ UCMSCs/day), with each visit 1–4 days apart from the next visit. The UCMSCs were collected from afterbirth tissue after obtained full-term healthy births. There were improvements reported after one month 1 month in the EDSS scores (*P* < 0.03) with a mean reduction of 0.48 (SD = 1.49) after 1 month and a reduction of 0.68 after 1 year, in addition to improvements in bladder and bowel functions, and sexual dysfunction (*P* < 0.01), in walk times (*P* < 0.02), and improved quality of life. MRI scans of the brain and cervical spinal cord showed inactive lesions in 15 out of 18 subjects after 1 year. There were no reported serious adverse events, with only headache or fatigue reported. This treatment is much better than MS drugs which have side effects and are much more costly. In addition, MS drugs are required to be taken daily or weekly [19].

## 6 Discussion

MS is an autoimmune disease and a complex disorder of the CNS which is associated with the loss of axons and long-term progressive disability that tends to be of an irreversible nature. There is no effective therapeutic method for this disease. Current medications can only manage and relieve the progress of the disease, but would not treat it thoroughly and cannot prevent recurrence. Recent studies have shown that cell-based therapies are able to repair CNS and can protect it against inflammatory responses caused by the immune system [57]. Cell-based therapies that rely on MSCs, which are multipotent cells with high self-renewal capabilities and immunomodulatory effects, have provided a new window for the prevention and treatment of different neurodegenerative diseases, such as MS, Parkinson's disease, Alzheimer's, and amyotrophic lateral sclerosis [41,45].

Many studies have suggested CD4^+^ cell involvement in the initiation and progression of MS by being auto-reactive and attacking the body's neurons. Once Th cells differentiate into Th1, Th2, or Th17 phenotypes, the new phenotypes have the ability to secrete special cytokines, including IFN γ, and TNF-α, which can promote inflammation. B lymphocytes are also linked to MS as TGF-β and TNF-α produced by these cells promote inflammation. In addition to these cells, CD8^+^ cells are also found in MS lesions. They play a role in the death of ODCs and CNS inflammation, and hence, the pathogenesis of MS. In a healthy individual who does not suffer from MS, Tregs play a crucial role in self-tolerance control and peripheral tolerance, by dynamically suppressing the activation and expansion of auto-reactive T cells and other immune cells in peripheral and CNS systems. Thus, reversing the function of Tregs can help to inhibit the proliferation of auto-reactive T cells and possibly stop MS progression [67,68].

There are many good types of MSCs, including BM-MSCs, ADMSCs, and UCMSCs. BM-MSCs have the ability to inhibit CD4^+^ and CD8^+^ T cell activation, can induce Tregs, and modulate macrophage phenotype switch from M1 to M2. Their actions result in the reduced production of pro-inflammatory cytokines [73]. Table 2 summarized studies in 5 countries on the use of BM-MSCs, the consensus being an improvement in the conduction of CNS pathways that are affected during MS, and generally a halt to MS progression in the patients. The results were confirmed using different methods, including MRI, volume of GEL, volume of BH, and EDSS scores. This places BM-MSCs as a novel therapeutic approach toward MS treatment [7]. The other type discussed is AD-MSCs, which are MSCs derived from human adipose tissue. Their source is abundant in quantity and extracting them has proven to be safe [11]. These cells have been used in clinical trials and stem cell research [30,59]. Table 3 has reported on their use in clinical trials run in 3 countries, the consensus being that AD-MSCs therapy is safe and it improves MS disabilities, including sexual problems and social activities in the patients studied.

Of the several types of MSCs, UCMSCs are the best option for MS treatment for several reasons. These cells can do a faster self-renewal than other MSCs, can differentiate into three germ layers, and can accumulate in damaged tissue or inflamed areas. They also have their own advantages that makes them the choice of MS therapy. First, the separation of the cells from the UC is easy, painless, and without ethical issues. Second, the amount of stem cells produced per unit area is high. Third, the cost of stem cell transfusion from the UC is not expensive. Fourth, these cells are very safe to use. Based on the studies presented in the section about UCMSCs, these cells would be considered as a safe and alternative option for treatment of the neurological parameters of MS, through results confirmed by EDSS, the nine-hole peg test, the expanded EDSS rating neurologic impairment, and the 25-foot walking time. UCMSCs have also been found to affect the function of the disabled Tregs in MS patients *in vitro* and revert them to normal conditions; impaired immunoregulatory function of Tregs constitute a main fixture of MS progression as being impaired means they cannot inhibit the proliferation of auto-effector T cells that would attack ODCs and initiate MS. Based on the information presented, it is the author's recommendation to emphasize the clinical utility of UCMSCs for regenerative medicine and immunotherapy.

## Consent for publication

The author grants the publisher the consent for publication.

## Data availability

The data for this work was collected from the references cited.

## Funding

There is no source of funding.

## Authors’ contribution

This article was written by a single author, Asma Alanazi. Email: as4asma2@hotmail.com.

## Declaration of competing interest

The author has no conflict of interest to declare.
